# Lac Vaccinum

**Published:** 1889-06

**Authors:** S. Swan

**Affiliations:** New York


### LAC VACCINUM.

S. SWAN, M. D., NEW YORK.

(This is *pure* milk potentized, *not* skim-milk, which is the *Lac Vaccinum Defloratum.*)

Miss H. took one dose 1M (Fincke). It caused frequent profuse discharge of clear urine, no sediment, and nearly colorless. White, watery leucorrhœa; pains in sacrum; sensation of plug in throat or larynx; sour taste in mouth; acid saliva staining handkerchief yellow; contractive pressing pain in stomach-pit, relieved by external pressure.

The following are cures from high potencies:

(1) After drinking fresh cow's milk in morning, had short rheumatic pains in knee and tarsal joints when walking; passage of stinking flatus. (Cured by Dr. Fincke.)

(2) Fever at night, followed by profuse sweat all over; the fever was preceded by chilly feeling, commencing at shoulders and then running up from feet to head; headache. *Lac vaccinum*$^{1200}$ cured.

(3) Mr. H. L. had ulcers on tongue, flat, white, sunken; tongue swollen, exceedingly sensitive, covered with white, slimy mucus on the parts not ulcerated; breath extremely fetid; sores extend to inside of cheeks and tonsils; deglutition so painful he refused to eat. *Mercurius* having had no effect, gave *Lac vaccinum*$^{30}$, a drop every four hours. Improvement commenced in an hour. In about six days brown crusts appeared at the corners of mouth, when the medicine was discontinued. In two weeks from the first the disease had entirely disappeared, leaving depressions on tongue where the ulcers had been, as if the surface had been eaten away. It was nearly three weeks before it resumed its natural appearance. The disease has never reappeared.

(4) Brown crusts, having a grêasy appearance, especially in corners of mouth, similar to what are called "butter-sores," yield rapidly to high potency of *Lac vaccinum*.

(5) Fullness of head as if too large and heavy.

(6) Vertigo.

(7) Eructations and passing of much flatus.

